# Visually guided respiratory motion management for Ethos adaptive radiotherapy

**DOI:** 10.1002/acm2.13441

**Published:** 2021-10-25

**Authors:** Taeho Kim, Zhen Ji, Benjamin Lewis, Eric Laugeman, Alex Price, Yao Hao, Geoffrey Hugo, Nels Knutson, Bin Cai, Hyun Kim, Lauren Henke

**Affiliations:** ^1^ Department of Radiation Oncology Washington University School of Medicine Saint Louis Missouri USA; ^2^ Department of Radiation Oncology University of Texas Southwestern Medical Center Dallas Texas USA

**Keywords:** adaptive radiotherapy, Ethos, respiratory motion management, visual guidance

## Abstract

**Purpose:**

Ethos adaptive radiotherapy (ART) is emerging with AI‐enhanced adaptive planning and high‐quality cone‐beam computed tomography (CBCT). Although a respiratory motion management solution is critical for reducing motion artifacts on abdominothoracic CBCT and improving tumor motion control during beam delivery, our institutional Ethos system has not incorporated a commercial solution. Here we developed an institutional visually guided respiratory motion management system to coach patients in regular breathing or breath hold during intrafractional CBCT scans and beam delivery with Ethos ART.

**Methods:**

The institutional visual‐guidance respiratory motion management system has three components: (1) a respiratory motion detection system, (2) an in‐room display system, and (3) a respiratory motion trace management software. Each component has been developed and implemented in the clinical Ethos ART workflow. The applicability of the solution was demonstrated in installation, routine QA, and clinical workflow.

**Results:**

An air pressure sensor has been utilized to detect patient respiratory motion in real time. Either a commercial or in‐house software handled respiratory motion trace display, collection and visualization for operators, and visual guidance for patients. An extended screen and a projector on an adjustable stand were installed as the in‐room visual guidance solution for the closed‐bore ring gantry medical linear accelerator utilized by Ethos. Consistent respiratory motion traces and organ positions on intrafractional CBCTs demonstrated the clinical suitability of the proposed solution in Ethos ART.

**Conclusion:**

The study demonstrated the utilization of an institutional visually guided respiratory motion management system for Ethos ART. The proposed solution can be easily applied for Ethos ART and adapted for use with any closed bore‐type system, such as computed tomography and magnetic resonance imaging, through incorporation with appropriate respiratory motion sensors.

## 1 INTRODUCTION

Ethos (Varian Medical Systems, CA, USA) adaptive radiotherapy (ART) is emerging with AI‐enhanced adaptive planning and high‐quality cone‐beam computed tomography (CBCT).^1^, ^2^, ^3^ CBCT‐guided online ART can compensate for interfractional changes of patient anatomy. For intrafractional changes, respiratory motion management is a critical element to reduce motion‐related image artifacts on abdominothoracic CBCT and improve tumor motion control during beam delivery.^4^ To the authors’ knowledge, however, Ethos systems have not incorporated a commercial solution for respiratory motion management.

Visually guided respiratory motion management has been introduced and implemented for some clinical procedures in radiotherapy.^5^, ^6^, ^7^ Although visual guidance is beneficial for multiple respiratory motion management methods in imaging and radiotherapy, providing visual guidance to the patient becomes cumbersome in closed bore‐type systems. For example, goggle‐based visual guidance requires sanitary covers, goggle storage space, and vision‐correcting lenses.^8^, ^9^ Electronic goggles require power and video signal wires fed into the bore, which can become trapped in moving parts and present a tripping hazard to patients and staff. Mounted monitor‐based approaches do not need sanitary covers but wiring and space‐related issues in the bore remain burdensome.^10^, ^11^ Reflecting mirrors with a screen outside the bore remove wires from inside the bore but adjusting mirrors and vision‐correcting lenses for patient specific care can increase procedure time.^12^, ^13^ Also, all of the above approaches require storage for patient‐related components such as goggles, sanitary covers, mirrors, corrective lenses, and monitors. Recently, digital projection systems have been utilized for closed bore‐type systems by directly displaying guiding images inside the long bore of magnetic resonance imaging (MRI) systems.^5^, ^14^

In this study, we propose an institutional visually guided respiratory motion management system to coach patients in regular breathing or breath hold during CBCT scans and manually gated beam delivery in Ethos ART. It consists of three components: (1) a respiratory motion detection system, (2) an in‐room display system, and (3) a respiratory motion trace management software. Through the study, we implemented the proposed system in a clinical Ethos ART workflow and demonstrated its applicability for respiratory motion control in CBCT scans and beam delivery.

## 2 METHODS

### 2.1 Respiratory motion detection system

**FIGURE 1:**
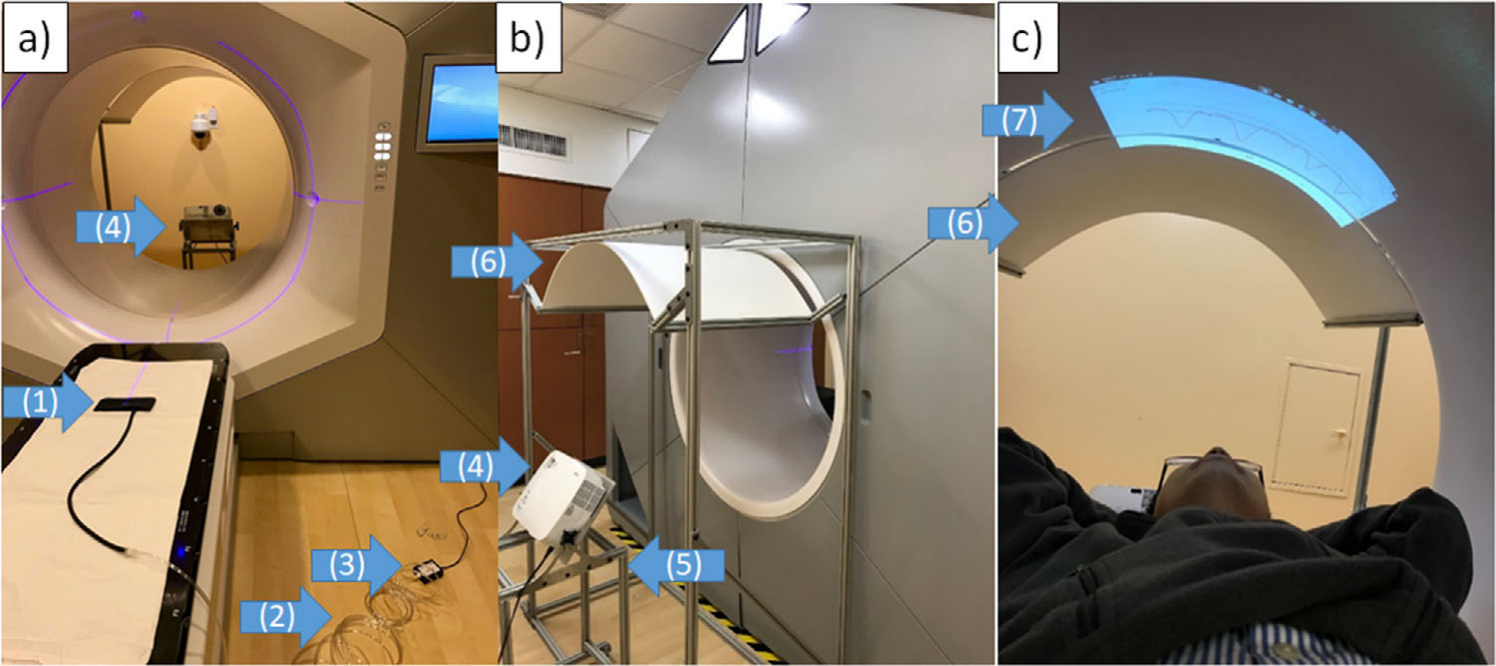
Components of the visually guided respiratory motion management system: (a) respiratory motion sensor and interface, (b) in‐room visual display system, and (c) visual guidance displayed on the Ethos bore; the components are: (1) air bag, (2) air tube, (3) pressure sensor, (4) projector, (5) adjustable stand, (6) extension screen, and (7) visual guidance display

Monitoring respiratory motion was accomplished via a pressure sensor and its connecting devices, as shown in Figure 1a. The hardware consisted of an air bag placed on patient abdomen with a Velcro belt, connected to a pressure sensor (Vernier, OR, USA). The pressure sensor measured the pressure changes from patient abdominal displacement against the Velcro belt when the patient was breathing. Respiratory signal was transferred to a computer system via a USB interface device (Vernier, OR, USA).

### 2.2 In‐room display system

The in‐room display system had two key components: an extended screen and a projector on an adjustable stand, as shown in Figure 1b. Displaying guiding images inside the bore of the system from the projector removed all issues with conventional visual guidance approaches, as previously described. For abdominothoracic sites, the projection area was increased using the extension screen so that the full length and width of the projected image could be utilized for visual guidance. Additionally, projection location can be adjusted using the adjustable stand, which makes this solution applicable for various patient habitus, as shown in Figure 1c.

### 2.3 Respiratory motion trace management software

**FIGURE 2:**
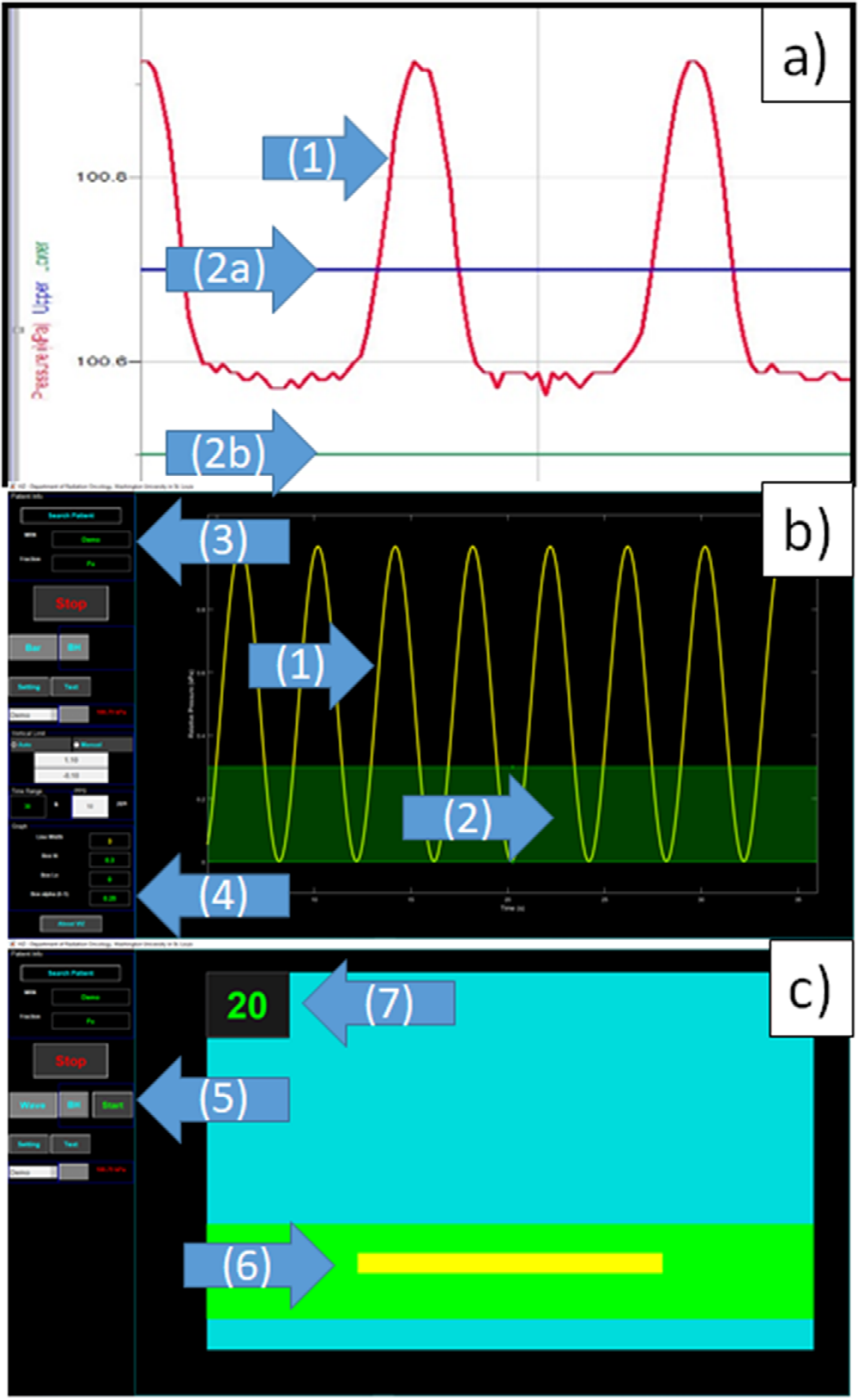
Components of respiratory motion trace management systems: (a) Logger Pro and (b & c) in‐house visual guidance software with (b) wave mode and (c) bar mode; the components are: (1) respiratory motion trace of the wave mode, (2) guiding window (2a: upper boundary and 2b: lower boundary), (3) patient information, (4) display setup menu, (5) display mode selection, (6) respiratory motion trace of the bar mode, and (7) breath‐hold timer

Respiratory motion signal was collected and displayed using either the LoggerPro software V3.14 (Vernier Software and Technology, Beaverton, OR, USA)^7^ or in‐house software, as shown in Figure 2. The LoggerPro software provided real‐time data collection and display (Figure 2a). The respiratory trace was displayed as a continuous wave with a guiding window defined by two horizontal lines that were adjustable for predetermined respiratory motion management, such as a full window for free breathing or a narrow window for breath hold.

In Figure 2b,c, the in‐house software display windows are shown, and it provided real‐time data collection and display. The display style can be switched between a wave mode and a bar mode. The wave mode showed the breathing signal as a continuous wave, while the bar mode displayed a horizontal bar moving up and down corresponding to the patient's breathing position. The real‐time data display region also included a breath‐hold timer to guide the patient during breath‐hold activity, as shown in Figure 2c. The display mode can be selected based on respiratory management methods in addition to patients’ vision and preferences on visual guidance display. According to our institutional experience, the wave mode was more useful for regular breathing and the bar mode for consistent breath holds. In our current implementation, however, we employed the wave mode to provide similar visual guidance to the patients and the clinical team's view with the LoggerPro software.

### 2.4 Visually guided respiratory motion management in Ethos ART

For demonstrating feasibility, three Ethos ART cases with our visually guided respiratory motion management system in place were assessed. Each patient had five fractions of ART. Patient 1 received treatment to a mobile retroperitoneal lymph node (LN) lesion using exhalation breath hold. Patients 2 and 3 received treatment to minimally mobile para‐aortic LN and retroperitoneal LN lesion, respectively. Since Patient 2 and 3 lesions were minimally mobile, they were treated without breath hold. In each fraction, clinical ART workflow consisted of (1) CBCT, (2) adaptive planning, (3) verifying CBCT and (4) arc delivery. If the adaptive plan had multiple arcs, we acquired a CBCT before each arc delivery.

Respiratory motion trace and couch shifts between intrafractional CBCTs were analyzed to evaluate respiratory motion management during ART. Respiratory motion trace analysis included selections of breathing time range and automatic breathing period detection. Amplitude and period variations (AV and PV, respectively) of each respiratory cycle were calculated from the average respiratory cycle using the in‐house software developed using MATLAB (Mathworks, MA, USA). To assess breath‐hold management, AV from the average breath‐hold trace and minimum breath‐hold time in all breath holds were calculated. In analysis, the amplitude was normalized to that of the average respiratory cycle. The couch shifts of each CBCT from the planning CBCT were manually recorded after CBCT registration. The compiled couch shifts are reported in this study.

## 3 RESULTS

### 3.1 Installation and routine QA

A standalone computer (visual guidance (VG) computer) in the console area was equipped for visual guidance. The respiratory motion trace management software on the VG computer was mirrored to the in‐room display system during routine QA and ART. Routine QA was performed on a daily basis to check the functionality of the pressure sensor, the VG computer, and the in‐room display system. The functional testing of the pressure sensor included an initial pressure reading (∼100 kPa: 101.325 kPa for one standard atmosphere) without any load and continuous pressure readings with some force applied by hand. The initial pressure reading remained ∼100 kPa depending on the daily atmospheric pressure. The continuous pressure readings confirmed the functionality of the pressure sensor. We utilized pressure changes to detect respiratory motion, and establishing a relative correlation between pressure in kPa and respiratory motion was considered in the functional testing. We understood that this sensor had some limitations, such as variation of the pressure baseline depending on patient setup and daily ambient pressure.

The extended screen system was used only for Ethos ART. The extended screen and the projector on the adjustable stand were retracted to the wall during Ethos image‐guided radiotherapy (IGRT).

### 3.2 Visually guided respiratory motion management in Ethos ART

Three Ethos ART cases treated with our visually guided respiratory motion management system in place are summarized in Table 1. In Ethos ART, respiratory motion management was applied as a standard of care while visual guidance was employed to assist the respiratory motion management. Each patient received patient‐specific respiratory motion management regardless of the employment of visual guidance, as listed in Table 1. For the first two fractions of Patient 2, the initial CBCT was taken with shallow breathing. However, significant motion artifacts degraded target and organ‐at‐risk visibility. Initial CBCT acquisition under breath hold was adopted for adaptive planning regardless of treatment motion management for the best image quality.

**TABLE 1:** Summary of respiratory motion management techniques for three patients undergoing Ethos adaptive radiotherapy; P1 includes the number of EBHs

	Fx1	Fx2	Fx3	Fx4	Fx5
P1	VG‐EBH (21)	VG‐EBH (18)	VG‐EBH (16)	VG‐EBH (15)	VG‐EBH (13)
P2	VG‐SB	VG‐SB	VG‐EBH/VG‐SB	VG‐EBH/VG‐SB	VG‐EBH/VG‐SB
P3	VG‐EBH/VG‐FB	VG‐EBH/VG‐FB	VG‐EBH/VG‐FB	VG‐EBH/VG‐FB	VG‐EBH/VG‐FB

Abbreviations: P, patient; VG‐EBH, visual guidance with exhalation breath hold; VG‐FB, visual guidance with free breathing; VG‐SB, visual guidance with shallow breathing.

**FIGURE 3:**
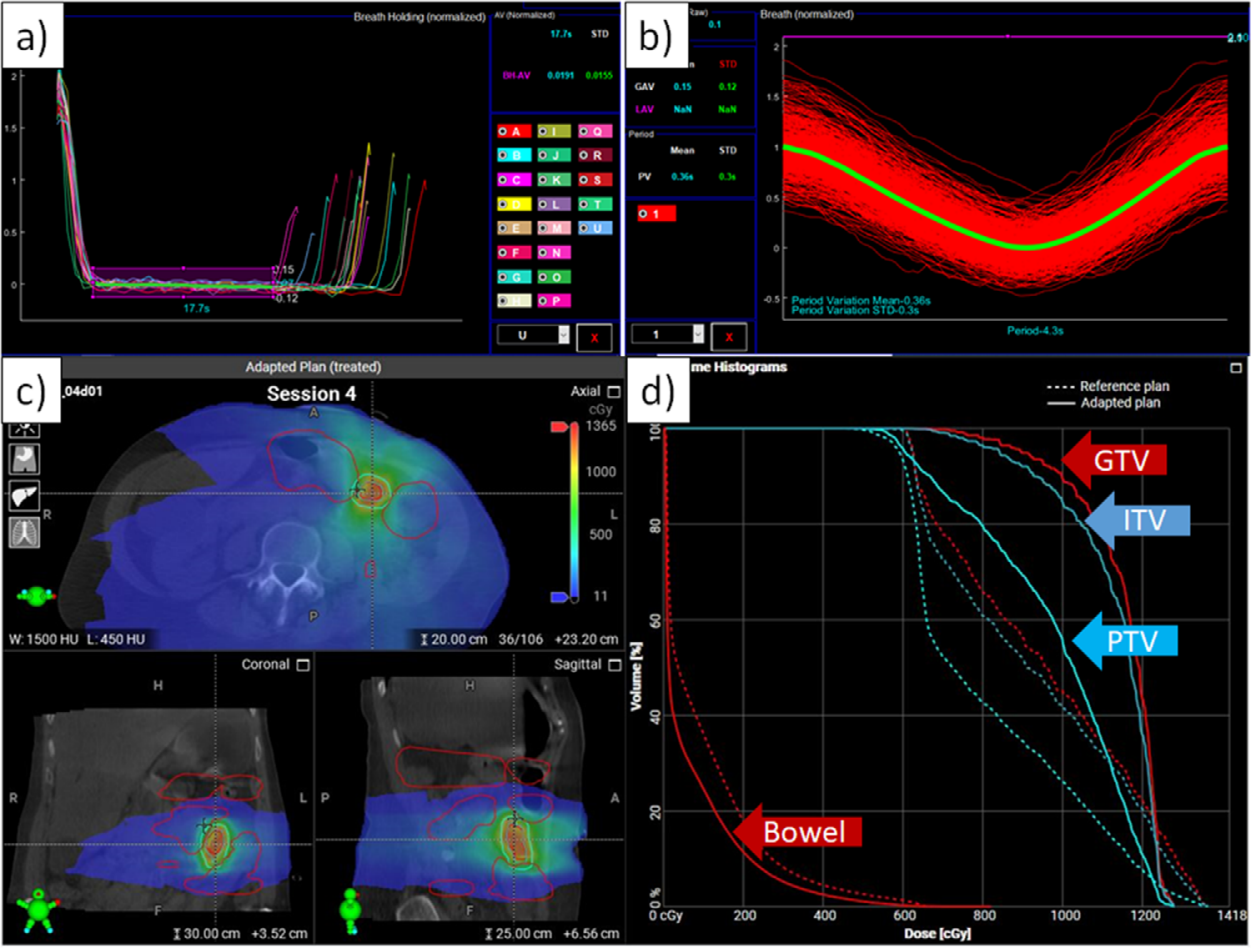
Example of respiratory trace analyses: (a) breath holds of P1 and (b) aligned (red curves) and average (yellow curve) free‐breathing traces of P3; (c) dose map on cone‐beam computed tomography (CBCT) and (d) dose volume histogram of an adapted plan for P1 are presented (dotted lines from the reference plan and solid lines from the adapted plan)

Figure 3 presents example respiratory trace analyses using the in‐house software—(a) breath‐hold traces and (b) free‐breathing traces. For instance, Figure 3a shows aligned breath‐hold traces to calculate AV from the average breath‐hold trace and minimum breath‐hold time in all breath holds. As a similar approach, Figure 3b presents aligned breathing traces (red curves) to determine AV and PV of each respiratory cycle from the average respiratory cycle (yellow curve). Figure 3b shows mean respiratory period ± standard deviation (4.3 ± 0.3 s) and mean respiratory AV ± standard deviation normalized by the respiratory amplitude of the average respiratory cycle (15% ± 12%).

Respiratory motion traces from three patients are presented in Table 2. The results from P1 include the respiratory amplitude of the average respiratory cycle in kPa, breath‐hold AV normalized by the respiratory amplitude of the average respiratory cycle (normalized breath‐hold AV), and minimum breath‐hold time in seconds since ART delivery was conducted with exhalation breath holds. The average minimum breath‐hold time is 21.5 s and the average AV during breath holds is 6.3% of the average respiratory cycle. Meanwhile, the results from P2 and P3 include the respiratory amplitude of the average respiratory cycle in kPa, respiratory AV normalized by the respiratory amplitude of the average respiratory cycle (normalized respiratory AV), and the respiratory period in seconds since ART delivery was conducted during free breathing. For P2, the average AV during shallow breathing is 25% of the averaged respiratory cycle (respiratory period: 3.5 ± 0.3 s). For P3, the average AV during free breathing is 19% of the average respiratory cycle (respiratory period: 3.9 ± 0.3 s).

**TABLE 2:** Summary of respiratory motion traces for three patients treated with Ethos adaptive radiotherapy; P1 includes the respiratory amplitude of the average respiratory cycle in kPa, breath‐hold amplitude variation normalized by the respiratory amplitude of the average respiratory cycle (normalized breath‐hold amplitude variation), and minimum breath‐hold time in seconds. P2 and P3 include the respiratory amplitude of the average respiratory cycle in kPa, respiratory amplitude variation normalized by the respiratory amplitude of the average respiratory cycle (normalized respiratory amplitude variation), and respiratory period in seconds

	Fx1	Fx2	Fx3	Fx4	Fx5	Mean ± Std
P1	0.44 kPa	0.22 kPa	0.22 kPa	0.16 kPa	0.11 kPa	0.23 ± 0.13 kPa
	1.9 ± 1.6 (%)	6.4 ± 5.5 (%)	5.4 ± 4.2 (%)	7.8 ± 6.1 (%)	9.9 ± 7.5 (%)	6.3 ± 3.0 (%)
	17.7 s	23.7 s	20.7 s	21.8 s	23.5 s	21.5 ± 2.4 s
P2	0.17 kPa	0.16 kPa	0.069 kPa	0.15 kPa	0.16 kPa	0.14 ± 0.04 kPa
	31 ± 28 (%)	22 ± 25 (%)	28 ± 25 (%)	18 ± 13 (%)	26 ± 22 (%)	25 ± 5 (%)
	3.9 ± 0.94 s	3.1 ± 0.65 s	3.5 ± 0.78 s	3.3 ± 0.34 s	3.6 ± 0.72 s	3.5 ± 0.3 s
P3	0.1 kPa	0.037 kPa	0.044 kPa	0.14 kPa	0.14 kPa	0.09 ± 0.05 kPa
	15 ± 12 (%)	24 ± 18 (%)	21 ± 15 (%)	20 ± 13 (%)	16 ± 13 (%)	19 ± 4 (%)
	4.3 ± 0.47 s	3.6 ± 0.50 s	4.1 ± 0.75 s	3.7 ± 0.46 s	3.9 ± 0.55 s	3.9 ± 0.3 s

### 3.3 Evaluation of anatomy positions on intrafractional CBCTs

Figure 3c,d shows a dose map on CBCT images and the dose volume histogram of the adapted plan (dotted lines from the reference plan and solid lines from the adapted plan). As presented, interfractional anatomy changes were considered in plan adaptation, resulting in improved coverage in target volumes and reduced dose to critical structures. After adapted plans were reviewed and approved, intrafractional anatomy changes were monitored using CBCTs before beam delivery while respiratory motion management was conducted.

**FIGURE 4:**
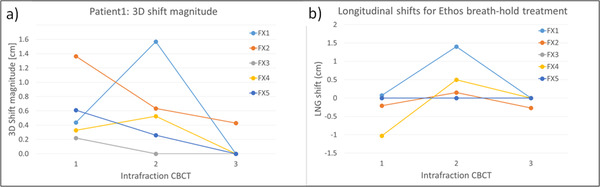
Intrafractional shifts of Patient 1 during Ethos ART: (a) 3D shift magnitude and (b) longitudinal shifts for Ethos ART with breath holds; 3D shift magnitude = $\sqrt{x^{2}+y^{2}+z^{2}}$ (x = lateral, y = vertical, and z = longitudinal shift in cm)

Table 3 presents intrafractional CBCT shifts of the three patients. The first verified CBCT shifts (shaded) were applied after adapted plan generation on the initial CBCT, before treatment delivery. The average time between the initial CBCT to the first verification CBCT for the three patients are 36, 24, and 26 min, respectively. Figure 4 shows intrafractional shifts between intrafraction CBCTs and the initial CBCT used for planning for Patient 1 during Ethos ART. Figure 4a shows 3D shift magnitude and Figure 4b presents longitudinal shifts for Ethos ART with breath holds. In FX1, a 1.4‐cm longitudinal shift at the second breath‐hold CBCT implies that the patient underwent a global shift during the long adaptive planning procedure. Nevertheless, the first verification CBCT shifts for P1 were noticeable, but the following CBCT shifts were reduced in the remaining fractions, indicating efficient intrafractional motion management, as shown in Figure 4. The large shift (other than the global shift in FX1) could have occurred due to residual respiratory motion in breath holds and internal organ motion, although respiratory motion was managed using the respiratory motion management system. P2 and P3 show negligible CBCT shifts (<1 mm).

**TABLE 3:** Summary of intrafractional cone‐beam computed tomography (CBCT) shifts (lateral, vertical, and longitudinal in centimeters) from three patients treated with Ethos adaptive radiotherapy; the first verification CBCT shifts (shaded) were applied after adapted plan generation on the initial CBCT. – Indicates no CBCT

	Shift#	Fx1	Fx2	Fx3	Fx4	Fx5
P1	1a	0.33, −0.28, 0.07	1.27, −0.45, −0.21	0.22, 0, 0	0.32, 0, 0.08	0.61, 0, 0
	1b	–	0, 0, 0	–	0.2, 0.36, −1.03	–
	2	0.56, −0.43, 1.4	−0.52, 0.33, 0.15	0, 0, 0	−0.15, −0.05, 0.5	−0.26, 0, 0
	3	0, 0, 0	0.3, −0.15, −0.27	0, 0, 0	0, 0, 0	0, 0, 0
P2	1	0, 0, 0	0, 0, 0	0, 0, 0	0, 0, 0	0, 0, 0
	2	0, 0, 0	0, 0, 0	0, 0, 0	0, 0, 0	0, 0, 0
P3	1a	0, 0, 0	0, 0, 0	0, 0, 0	0, 0, 0	−0.06, 0.06, 0
	1b	0, 0, 0	–	–	–	–
	2	0, 0, 0	0, 0, 0	0, 0, 0	0, 0, 0	0, 0, 0

## 4 DISCUSSION

This study presented our institutional visually guided respiratory motion management solution for Ethos ART. It can be easily applied and personalized for Ethos radiotherapy and extended to any closed bore‐type system through incorporating other respiratory motion sensors.

The potential benefits of the proposed solution include its simplicity and flexibility for closed bore‐type systems. The three components of the system are simple to equip using institutional resources. The projection screen position and size can be easily adjusted on the extended screen for both upper torso and pelvic treatment sites. Sanitary covers, device storage, and electric wires inside the bore were no longer needed. In addition, the three components can be replaced with similar items. For example, the extended screen can be replaced with a thin projection screen attached to the patient couch.^15^ In our institutional setup, an inflation bag for a blood pressure cuff (MDF instruments, PR, USA) was utilized, instead of a respiratory air bag, which was connected to the pressure sensor (radiolucent: 0.02% attenuation).^7^ Furthermore, LoggerPro software was easily employed with our in‐house solution while data analysis was completed using another software (Microsoft Excel or MATLAB). Alternatively, we developed in‐house software to improve the features of the LoggerPro software, including data analysis, while keeping the main features of the visual guidance system consistent.

When the proposed solution was initially implemented into the clinical workflow, the LoggerPro software was used as the respiratory motion trace management software since it communicated with the pressure sensor for data collection. The basic function of the visual guidance system was performed by displaying the respiratory trace and the guiding window. However, the LoggerPro software had limited features, resulting in the development of our own in‐house software. For example, the developed software had two display modes, visual effect adjustments for the respiratory trace and the guiding window, and a breath‐hold timer. In addition, the in‐house software has a breathing pattern analyzer. Analysis of breathing motion included selection of breathing time range and automatic breathing period detection. Parameters such as AV and PV can be calculated. An adjustable window on the pattern analysis graph was used to select portions of the breathing pattern to calculate local AV, suggesting a range for the guiding window. The breath‐hold analyzer also included time‐range selection. An adjustable window was also overlaid on the breath‐hold analysis plot to calculate AV to determine an achievable breath‐hold time.

The proposed solution has a few limitations. First, since it utilized pressure changes to detect the respiratory motion, interfractional variation of the pressure baseline was unavoidable due to variations in patient setup and daily ambient pressure. For example, the pressure baseline can be changed based on the tightness of the Velcro belt against the pressure sensor (intersetup baseline change) in addition to the daily atmospheric pressure (daily baseline change). In intrafractional motion management, the interfractional change of the pressure baseline was not an issue since the pressure sensor measured the pressure changes from the initial pressure baseline after the setup. However, an insecure setup can cause a drift of the initial pressure baseline, so we secured the Velcro belt against the pressure sensor. Second, a metal frame was used to support the extended screen. Since Ethos has a safety interlock on its cover, setup of the extended screen requires additional caution. A thin screen attached to the patient couch can be an alternative solution for this limitation. Third, the proposed solution did not have direct beam control. Since our institutional Ethos did not have an automatic gating function, beam gating was controlled manually by an operator. Visually guided respiratory motion management became very useful in our manually gated treatment by coaching the patent in regular breathing or breath hold while simultaneously displaying the patient's real time respiratory motion to the operator.

The efficacy of the proposed solution is presented through evaluating respiratory motion traces in Table 2 and intrafractional shifts based on tumor positions on CBCTs in Table 3. The study demonstrated the clinical suitability of the proposed solution in Ethos ART.

## 5 CONCLUSION

The study demonstrated the institutional visually guided respiratory motion management system for Ethos ART. The proposed solution can be easily applied for Ethos ART and extended to any closed bore‐type systems, such as computed tomography and MRI, through incorporating appropriate respiratory motion sensors.

## AUTHOR CONTRIBUTIONS

Taeho Kim, Zhen Ji, Benjamin Lewis, and Bin Cai contributed to development of the systems, data collection, analysis, and writing of the manuscript. Eric Laugeman, Alex Price, Yao Hao, Geoffrey Hugo, Nels Knutson, Hyun Kim, and Lauren Henke contributed to data collection and design of data analysis, and provided clinical assistance. All authors discussed the results and contributed to the final manuscript.

## Data Availability

The data that support the findings of this study are available from the corresponding author upon reasonable request.
